# Unilateral Cleft Lip and Palate Has Asymmetry of Bony Orbits: A Retrospective Study

**DOI:** 10.3390/jpm13071067

**Published:** 2023-06-29

**Authors:** Eeva Kormi, Elina Peltola, Niilo Lusila, Arja Heliövaara, Junnu Leikola, Juho Suojanen

**Affiliations:** 1 Päijät-Häme Joint Authority for Health and Wellbeing, Department of Oral and Maxillofacial Surgery, Päijät-Häme Central Hospital, 15850 Lahti, Finland; 2 HUS Diagnostic Center, Radiology, Helsinki University Hospital, 00029 Helsinki, Finland; 3 Päijät-Häme Joint Authority for Health and Wellbeing, Department of Radiology, Päijät-Häme Central Hospital, 15850 Lahti, Finland; 4 Cleft Palate and Craniofacial Centre, Department of Plastic Surgery, Helsinki University Hospital, 00029 Helsinki, Finland; 5 Clinicum, Faculty of Medicine, University of Helsinki, 00014 Helsinki, Finland

**Keywords:** cleft palate, cleft lip, facial asymmetry, orbit, surgery, computer-assisted, orthognathic surgery

## Abstract

Facial asymmetry is common in unilateral clefts. Since virtual surgical planning (VSP) is becoming more common and automated segmentation is utilized more often, the position and asymmetry of the orbits can affect the design outcome. The aim of this study is to evaluate whether non-syndromic unilateral cleft lip and palate (UCLP) patients requiring orthognathic surgery have asymmetry of the bony orbits. Retrospectively, we analyzed the preoperative cone-beam computed tomography (CBCT) or computed tomography (CT) data of UCLP (*n* = 15) patients scheduled for a Le Fort 1 (*n* = 10) or bimaxillary osteotomy (*n* = 5) with VSP at the Cleft Palate and Craniofacial Center, Helsinki University Hospital. The width, height, and depth of the bony orbit and the distance between the sella turcica and infraorbital canal were measured. A volumetric analysis of the orbits was also performed. The measurements were tested for distribution, and the cleft side and the contralateral side were compared statistically with a two-sided paired *t*-test. To assess asymmetry in the non-cleft population, we performed the same measurements of skeletal class III patients undergoing orthognathic surgery at Päijät-Häme Central Hospital (*n* = 16). The volume of bony orbit was statistically significantly smaller (*p* = 0.014), the distance from the infraorbital canal to sella turcica was shorter (*p* = 0.019), and the anatomical location of the orbit was more medio-posterior on the cleft side than on the contralateral side. The non-cleft group showed no statistically significant asymmetry in any measurements. According to these preliminary results, UCLP patients undergoing orthognathic surgery show asymmetry of the bony orbit not seen in skeletal class III patients without a cleft. This should be considered in VSP for the correction of maxillary hypoplasia and facial asymmetry in patients with UCLP.

## 1. Introduction

Unilateral cleft lip and palate (UCLP) is a common congenital developmental malformation, where the upper lip fails to fuse to the maxillary prominence and palatal sides fail to merge, causing congenital fissure. The prevalence of cleft lip and palate is 0.45 in every 1000 live births [1].

The treatment goals of UCLP are good speech, hearing, maxillary growth, facial aesthetics, and symmetry, as well as psychosocial well-being. However, even with multidisciplinary cleft teams and skillful surgery, orthognathic surgery is often needed for the correction of crossbite, maxillary hypoplasia, and asymmetry. The need for orthognathic surgery varies according to the type and extent of cleft. The need is 15.6–50.4% in patients with UCLP [2,3,4,5,6,7,8].

Asymmetry can be an anatomic, functional, and aesthetic challenge in unilateral clefts. Maxillary skeletal and dental differences and asymmetry between the cleft and non-cleft sides have been well-studied in 2D roentgenologic [9,10,11] and 3D evaluations [12,13]. However, the literature on asymmetries and irregularities of bony orbits in unilateral clefts is scarce. Hypertelorism and orbital eye fissure length asymmetry have been reported in anthropometric studies of patients with UCLP [14]. With the increased use of 3D imaging methods, skeletal asymmetry has been evaluated more in detail, as well as in the orbital, zygomatic, and frontal bones [15,16,17]. Interestingly, cephalometric bony landmarks orbitale and orbital planes are often used in cephalometric analyses and reference planes when planning orthognathic surgery. These landmarks are also included in the Frankfort horizontal plane commonly used in analyzing lateral cephalograms, as well as in head-positioning in the virtual surgical planning of a 3D skull model [18].

Because of the challenging anatomy and multidimensional asymmetry in unilateral clefts, 2D cephalograms may provide insufficient data for preoperative planning regarding orthognathic surgery. Asymmetry can often be seen in lateral cephalograms as mandible lower border discrepancy, the blurring of the nasal spine due to the rotation of the maxillary midline, or the overlapping of upper incisors’ tips shadows [19]. Conventionally, antero-posterior skull X-rays have been used for evaluating the asymmetry together with cephalograms to predict possible transversal problems during surgery.

Over two decades, 3D models generated either by radiologists or clinicians have aided in assessing the bony midline asymmetry in severe cases [20]. Clinical analysis software for cephalometric analysis and 3D modeling, such as Dolphin (Patterson Dental, Saint Paul, MN, USA) or Romexis (Planmeca, Helsinki, Finland), are now commonly used in everyday orthodontics. However, their use is qualitative in nature and typically helps to visualize potential problems.

The use of preoperative computer-assisted design/computer-assisted manufacturing (CAD/CAM) 3D planning is becoming more common, in orthognathic surgery for patients with craniofacial deformities and clefts. The reports on virtual surgical planning in patients with cleft lip and palate have been promising [21,22,23]. Three-dimensional VSP is often used even though individualized CAD/CAM surgical tools are not used [24]. The 3D imaging data, which are used in virtual preoperative planning, can further be used during surgical follow-ups to compare the VSP with the actual result and for other research purposes, such as skeletal stability after surgery. The rationale for the study lies in the fact that we have noticed in our clinical practice that bony orbits can be asymmetrical in UCLP, which often makes it difficult to position the skull for VSP. The bony orbits are commonly used as landmarks in skull positioning prior to virtual planning [19]. Also, as emerging technologies may use semi-automated or automated segmentation in VSP, the asymmetry must be considered.

The aim of this study is to evaluate whether non-syndromic UCLP patients with maxillary hypoplasia and malocclusion and mandated orthognathic surgery have asymmetry of the bony orbits. The null hypothesis H_0_ was that unilateral clefts have symmetrical anatomies and bony orbits positioned between the cleft and the contralateral sides. The alternative hypothesis H_A_ was that orbits have asymmetry. To assess the relevance of the findings, we also performed the measurements in non-cleft skeletal class III and dental discrepancy patients undergoing orthognathic surgery.

## 2. Materials and Methods

This retrospective study examined patients with UCLP who were treated at the Cleft Palate and Craniofacial Center, Department of Plastic Surgery, Helsinki University Hospital and Helsinki University, Finland. The inclusion criteria comprised patients scheduled for a Le Fort 1 osteotomy or bimaxillary osteotomy surgery with preoperative CAD/CAM 3D virtual surgical planning (VSP) by the end of 2021. Patients with known syndromes affecting craniofacial structures were excluded.

To evaluate the relevance of the findings, we had non-cleft maxillary hypoplasia patients from Päijät-Häme Centra Hospital, Lahti, Finland. For this comparative group, the inclusion criteria comprised patients planning to undergo orthognathic surgery due to skeletal class III and dental discrepancies with preoperative VSP between 2018 and 2022.

**Figure 1:**
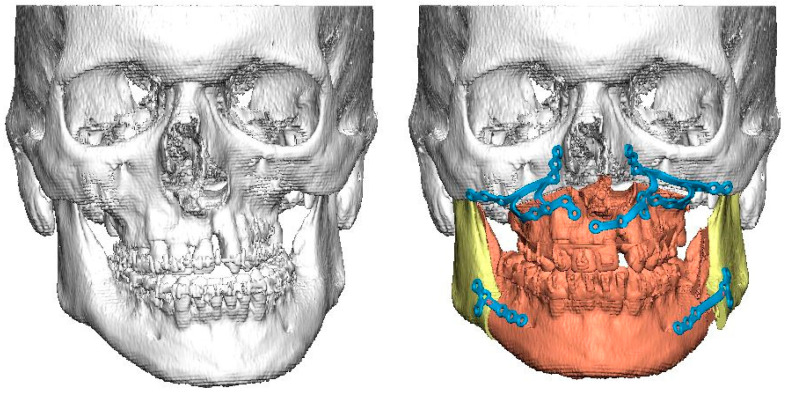
On the left is a preoperative surface model of a patient with UCLP and severe midline asymmetry. On the right is the planned correction of bony asymmetry with CAD/CAM-generated patient-specific osteosynthesis for simultaneous Le Fort I osteotomy and bilateral sagittal split osteotomies.

Medical charts from hospitals’ archives and databases were used for data collection. All patients had cone-beam computed tomography (CBCT) or computed tomography (CT) with a slice thickness of 1 mm or less and 0° gantry tilt taken for 3D planning purposes. The radiological anatomy of bony orbits was analyzed retrospectively from CT or CBCT imaging data that had been used in the virtual planning of orthognathic surgery and for CAD/CAM production of patient-specific surgical drill and cutting guides, as well as individualized osteosynthesis (Figure 1)

**Figure 2:**
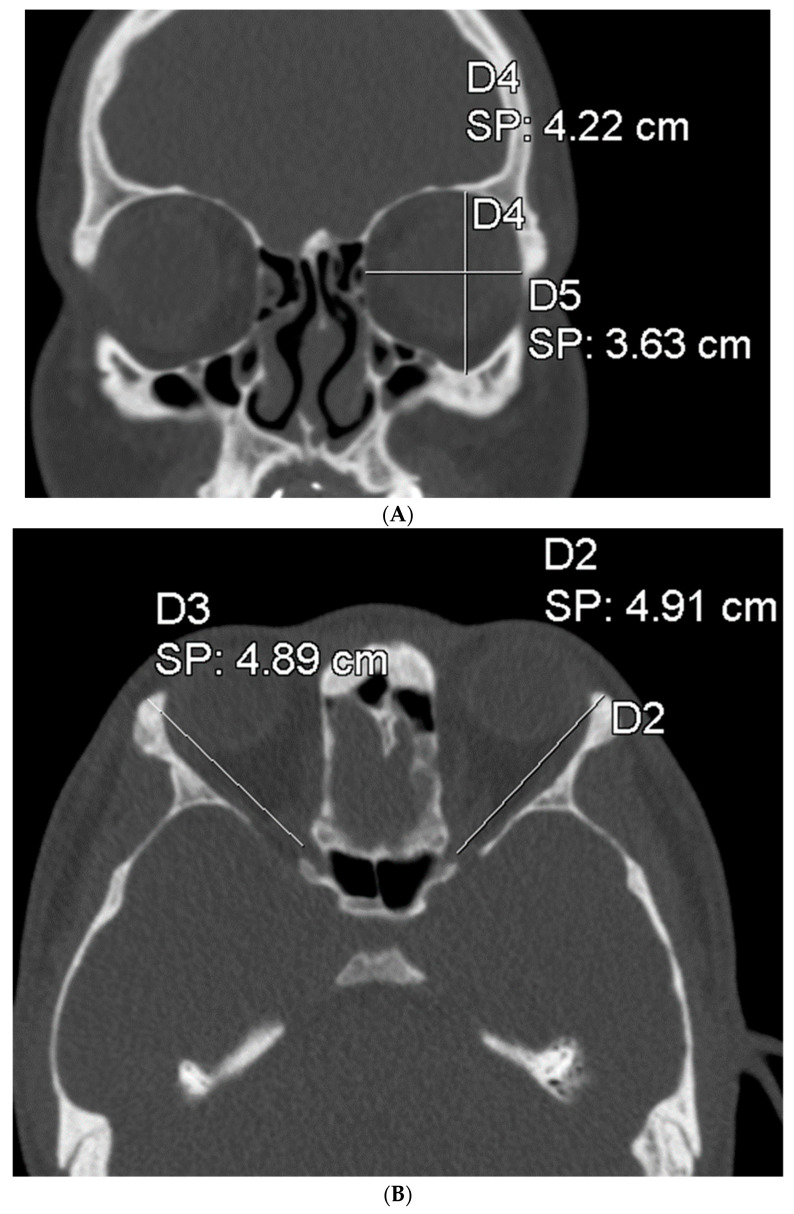

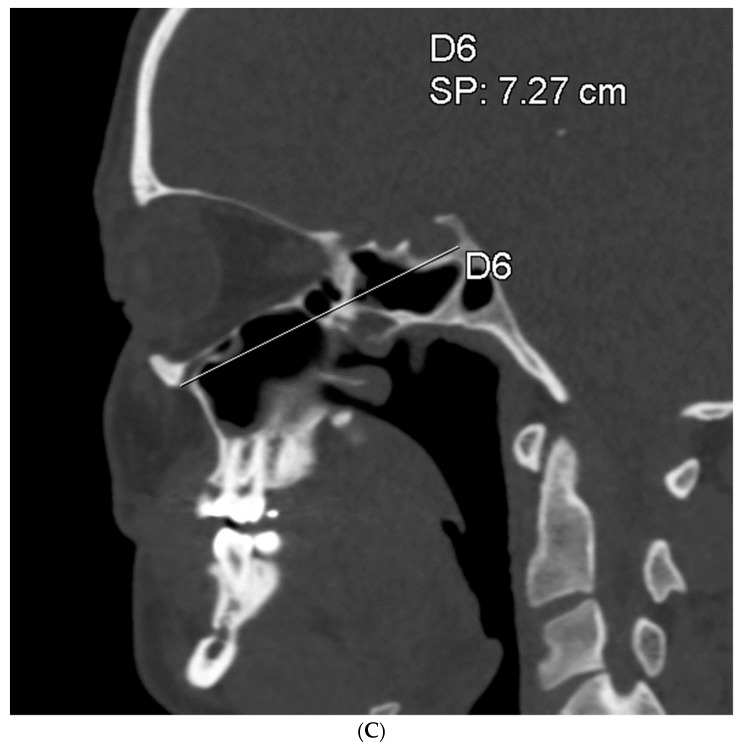
(**A**) The width and height of the bony orbit from the most anterior CBCT or CT slice showing the whole orbital rim. (**B**) The depth from the frontozygomatic suture to the optic canal opening. (**C**) The position of bony orbit was assessed as the distance of the cranio-lateral border of the infraorbital canal opening to the inner posterocaudal wall of sella turcica, measured from an oblique sagittal plane.

**Figure 3:**
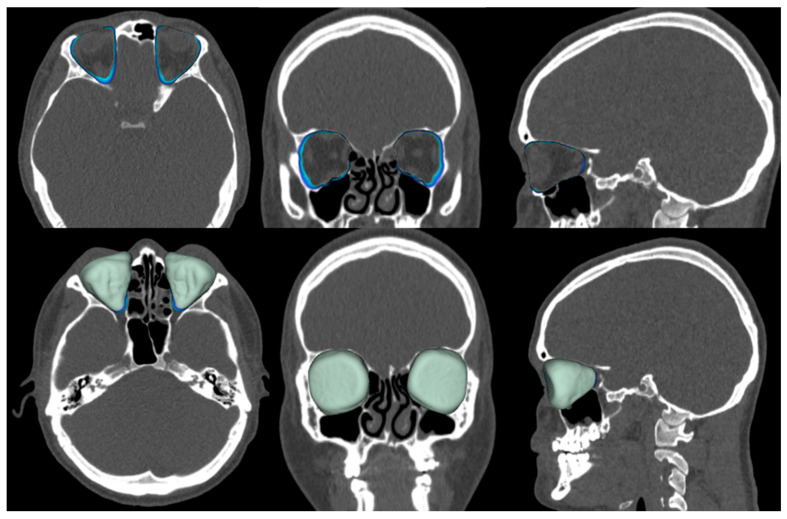
Assessment of the volume of the bony orbit in the cleft group. Top row contours, and bottom row colors.

To assess the size of the rim of bony orbit, the width and height were measured from the most anterior CBCT or CT slice showing the whole orbital rim (Figure 2A), and the depth was the distance from the frontozygomatic suture to the optic canal (Figure 2B). The position of bony orbit was assessed as the distance of the cranio-lateral border of the infraorbital canal opening to the inner postero-caudal wall of the sella turcica, measured from an oblique sagittal plane (Figure 2C). Helsinki University Hospital radiologist workstations and Syngo software (Siemens Healthineers, Erlangen Germany) were used for the analysis according to the manufacturer’s instructions for quantitative analysis. The volume of bony orbit was measured with semi-automated segmentation to avoid potential measurement-related bias since the cleft side is always visible to the radiologist in the lower sections during the orbital analysis. Semi-automated orbital volume analysis was performed using CMF Orbital Software (Disior Ltd., Helsinki, Finland). To use the software, the radiologist only selects a seed point inside the orbital vault and confirms the side to be examined. The apex of the orbit at the conjunction of the optic nerve and bulbus was used as a seed point in all cases. After selecting the seed point, the virtual program-generated triangle mesh iteratively expands from the starting position until it meets the bony walls of the orbit. The anterior expansion of the volume mesh network automatically stops when the mesh reaches the bony rim of the orbit (Figure 3). This system is described in more detail, and the reliability of the software measurements is proven to be high (0.992 (95% CI 0.987–0.997 intraobserver ICC and 0.989 (95% CI 0.983–0.993)) interobserver ICC in intact orbit) [25]. All volume analyses and the surface reliability of the software algorithm were confirmed by the radiologist in all cases. All the measures of the cleft patients were made by the same radiologist (E.P.).

To assess non-cleft group asymmetry, the same linear measurements for the cleft group were made by the radiologist (N.L.). Volume was measured as described earlier [26]. Briefly, measurements were made with manual segmentation by defining the contours of the orbit and using 3D segmentation and volume measurement tools. Päijät-Häme Central Hospital radiologist workstations and GE HealthCare AW Server software were used for the analysis according to the manufacturers’ instructions for quantitative analysis.

All the data were collected using Microsoft Excel software, from where it was moved to statistics software SPSS (IBM^®^, v.22, Armonk, NY, USA) for statistical analysis. Significance was set to 0.05. We tested the distribution of data with a Shapiro–Wilks test (Table 1). If the test did not show evidence of non-normality, we used a parametric test.

For the cleft group, the cleft side was compared to the contralateral side with a two-tailed paired *t*-test. For the non-cleft group, the same tests were made between the right and left sides.

For further analysis, we divided the cleft patients into Le Fort 1 and bimaxillary osteotomy subgroups, which were compared with the Mann–Whitney U-test for differences. To assess intra-rater reliability, 40 linear measurements were remeasured by the same radiologist blinded to previous results and tested with Cohen’s Kappa analysis.

## 3. Results

We found 17 patients with UCLP, and 2 were excluded because of known syndromes. Fifteen non-syndromic UCLP patients fulfilled the inclusion criteria and had sufficient CBCT or CT data to measure the orbital area. Of the 15 patients, 10 underwent Le Fort I osteotomy, and 5 underwent bimaxillary surgery osteotomy. Four radiographic measurements had to be partially excluded during the analysis because of unclear radiologic landmarks (three volume measures and one height and width measure).

Patient characteristics, cleft types, and information about the previous and planned operations are given in more detail in Table 2. Because of their severe maxillary hypoplasia and crossbites with functional, aesthetic, and/or social difficulties, two patients in this series underwent early maxillary osteotomies during growth. One patient with UCLP had ocular pathology (myopia and astigmatism).

Of the non-cleft group, 16 patients fulfilled the criteria and had sufficient data for analysis. Of them, two height and volume measurements and one width measurement were excluded because of inadequate radiological data.

We rejected our null hypothesis in the volume and position of the orbit (Table 3). The volume of bony orbit differs from the contralateral side (*p* = 0.014) and is smaller on the cleft side in all patients except for two, with a mean difference of 836 mm^3^. The mean distance between the infraorbital canal and sella turcica shows asymmetry as well (*p* = 0.019), and the anatomical location of the orbit is more medio-posterior on the cleft side than on the non-cleft side. However, the mean difference is less than 2 mm. There are no statistically significant differences in orbital height, width, and depth between the cleft and non-cleft sides.

For further analysis, we divided the cleft patients into two subgroups according to the type of planned surgery, bimaxillary surgery, or maxillary Le Fort 1 osteotomy. For each measure, we calculated the percent difference in each subgroup, and then the subgroups were compared with a Mann–Whitney U-test. A statistically significant difference was found between these subgroups in the position (Figure 2C) (*p* = 0.040) of the orbit (Table 4). The patients with planned bimaxillary surgery had more orbital asymmetry between the cleft and the non-cleft side than those with planned maxillary surgery.

The non-cleft group of class III patients had no statistically significant asymmetry in any of the measures (Table 5).

Intrarater reliability was analyzed with Cohen’s Kappa analysis. The radiologist remeasured a total of 40 linear measurements from five patients, blinded from previous measurements. The Kappa value was κ = 0.843, showing excellent agreement. The average difference in measurements was 0.015 mm, showing excellent accuracy.

## 4. Discussion

Our study shows that UCLP patients undergoing orthognathic treatment show asymmetry of the bony orbits, which is not seen in non-cleft skeletal class III orthognathic patients. The orbital volume is smaller, and the orbit is positioned more medio-posteriorly in our study population.

Slight asymmetries are often seen in the normal population; despite this, normally bony orbital volumes show no statistically significant difference, as seen in our non-cleft group, which parallels previous studies [27,28,29]. For this reason, the orbits have been adopted as part of bony landmarks for skull positioning before cephalometric analysis, as well as VSP. Our results on cleft patients parallel the study by Harikrishnan et al. [15], who found asymmetry not only in the maxilla but also in the orbital, zygomatic, and frontal bones. Asymmetry was measured from a 3D model of a UCLP skull from a patient’s cone-beam computed tomography. Patel et al. [16] studied 3D facial asymmetry with an asymmetry index in 25 subjects with UCLP. Facial asymmetry extended to involve the upper, middle, and lower facial skeleton. Most of these subjects had significant degrees of midfacial asymmetry, but there was individual variation. Maxillary, nasal, and orbital asymmetry (cranio-caudal displacement of infraorbital rim) has been observed in 3-month-old babies (*n* = 21) with UCLP [17]. The asymmetry corresponded to a dislocation of the maxillary segment on the cleft side [17]. In addition, retrusive suborbital projection of the orbitomalar region and hypertelorism were reported in patients with UCLP [14,30,31]. However, no strong evidence of a relationship between interorbital distance and cleft severity was found with the utilization of 3D surface imaging [32]. With numerous surgical techniques, as well as operating surgeons’ preferences differing to some extent, it is difficult to define which part of the asymmetry is related to the cleft and which part is iatrogenic, making the scientific evaluation difficult.

Interestingly, we found preoperative differences between the position of the orbit in patients with maxillary Le Fort 1 and bimaxillary osteotomy surgery. The patients who were to undergo bimaxillary surgery had more severe orbital asymmetry than those who were scheduled for only maxillary surgery. The choice between bimaxillary surgery, maxillary le Fort I surgery, or no surgery at all is based not only on the anatomy but also the preference of the orthodontists, surgeons, patient, and treatment goals. The number of patients referred to surgery is higher than the number who underwent orthognathic correction [8]. Bimaxillary surgery patients often have maxillary hypoplasia with canting of the occlusal plane, skeletal and dental midline asymmetries, and nasal deviation, although bimaxillary surgery may also be needed in severe anteroposterior discrepancy and bimaxillary retrusion [33].

In our present cohort, no ocular pathology was detected. Lilius [34] evaluated 1586 Finnish probands with clefts, of whom 345 (21.8%) had associated anomalies. Of the 268 probands with UCLP, 6 (2.2%) had ocular anomalies. Of the 267 probands with clefts of the lip or lip and alveolus, 4 (1.5%) had ocular anomalies. Anchlia et al. [30] reported ocular abnormalities in cleft lip and palate patients (*n* = 322) with and without syndromes. Orbital defects (hypotelorism and telecanthus) were found in 17% of patients [35]. In a large data collection from Texas Birth Defects, 21% of non-syndromic infants with cleft lips with or without palate (*n* = 5289) had at least one additional congenital anomaly. Eye anomalies were often combined with other defects [31].

Facial development involves a series of well-coordinated events. The face is formed by five prominences that appear in the fourth week: a frontonasal prominence and paired maxillary and mandibular prominences [36]. The medial and lateral nasal processes form within the frontonasal prominence during the fifth week, subsequently migrating and fusing in the midline to form the nose and the philtrum [37]. Cleft lip occurs when the medial nasal prominence and maxillary prominence fail to fuse. Cleft palate can occur in isolation when the palatal shelves fail to fuse in the midline or in combination with a cleft lip. The embryology of the orbit is linked to the facial development, and orbital asymmetry has also been described in other developmental defects. Patients with unilateral coronal synostosis (UCS) have persistent facial asymmetry at school age, with the greatest levels of asymmetry in the facial middle third, orbit, and nasal root [38]. In addition, orbital volume on the affected side has been reported to be significantly lower than on the non-affected side, although no association between the orbital volume ratio and severity of UCS was found [39]. In a 3D-surface scanning follow-up study, 90% of the patients with UCS (*n* = 22) had significant facial asymmetry throughout the facial area [40]. Marked orbital asymmetry has been linked to hemifacial microsomia [35]. Orbital volume was 10% smaller on the affected side in 80% of patients [41].

In addition to occlusion correction and the sagittal and vertical skeletal relationships in orthognathic surgery, correcting facial asymmetry is of major importance for patients with clefts [42]. Patients with UCLP may have asymmetry that extends to the upper midface and orbital level. This asymmetry can be difficult to quantify clinically and in the traditional 2D lateral cephalometric analysis. Asymmetry can affect the reliability of landmarks and reference planes that are used in 2D cephalometric analyses and surgical planning. The use of 3D virtual surgical planning and wafer-free surgery is increasing in orthognathic surgical planning, both in patients with [21,22,23] and without clefts [19,43,44]. The median deviation of VSP compared to the surgical outcome is assessed to be 0.39 mm [44], and navigation offers an accuracy of 0.5–1 mm, which is insufficient for bone segment positioning compared to what can be achieved with VSP and surgical guides [45]. Thus, even small differences matter. It is evident that the cleft itself or surgical and non-surgical management of the cleft can affect growth and lower midface asymmetry [46,47,48], but our results suggest that the effect on facial growth extends more cranially in facial bones. These findings may have clinical relevance if automated software is used for VSP since positioning the skull in a three-dimensional working space affects the movement of segmented jaws in the X, Y, and Z axes, thus affecting the achieved roll, pitch, and yaw of the maxilla in surgery. A few degrees of difference in the angle of the skull position prior to VSP can lead to notable midline asymmetry of soft tissues and the underlying bone. While the normal population has minor asymmetries, bony orbital volumes show no statistically significant difference [27,28,29]. It has been demonstrated earlier that even jaw asymmetries in the population needing orthodontic treatment are as rare as 5% in both pediatric and adult patients [49,50]. For this reason, orbital asymmetries are most likely sporadic anomalies or potentially undiagnosed congenital syndromes, such as mild hemifacial microsomia in non-cleft osteotomy patients.

With 3D imaging and planning, it improves accuracy in the evaluation and overall treatment plan for asymmetry, and potential skeletal and soft-tissue differences between the cleft side and contralateral side are easier to detect. With UCLP patients, we always adjust the skull position for VSP individually instead of trusting the automated position. However, the differences in the bony orbital area in our study were small, and the clinical relevance should be considered case by case.

The small number of patients is a major limitation of this preliminary study, and larger populations are needed to confirm the results. Another limitation is the selected study population, and the results may not be generalized to all cleft patients. Patients who are to have orthognathic surgery typically have crossbites and rather severe maxillomandibular skeletal discrepancies [19]. This data may be biased when compared to those patients with UCLP with good dental arch relationships and favorable maxillary growth. Moreover, we assessed only the orbital asymmetry, and the dysmorphology of the upper, middle, and lower face should be assessed as well. Utilizing the contralateral side of the patient as a control regarding the affected side in lieu of a separate control group should also be considered. It is possible that the contralateral side of the patients with unilateral clefts is also affected. Still, the cleft side seems to be constantly smaller, with shorter measurements overall. Although we had non-cleft patients as normal references, they were not treated in the same hospital; because the imaging and measuring protocols were not exactly similar, the non-cleft group could not be used as matched controls. Thus, our non-cleft group acts only as a reference to show that there is no statistically significant asymmetry in skeletal class III patients in general.

## 5. Conclusions

UCLP patients undergoing orthognathic treatment have asymmetry of the bony orbit in this preliminary study. The asymmetry is often relatively subtle. Even if these differences are small, they add to a broader understanding of the clinical findings regarding UCLP but may not be generalized to all cleft patients. However, this should be considered in orthognathic virtual surgical planning for the correction of maxillary hypoplasia and facial asymmetry in patients with UCLP, especially if orbit-related bony landmarks are further used for automated skull positioning prior to the computed segmentation.

## Figures and Tables

**Table 1:** Test (Shapiro–Wilk) for the distribution of data.

	Cleft Patients		
	W	df	Sig.
Cleft height	0.947	12	0.594
Contralat height	0.900	12	0.159
Cleft width	0.910	12	0.211
Contralat width	0.951	12	0.654
Cleft depth	0.942	12	0.520
Contralat depth	0.921	12	0.295
Cleft position	0.916	12	0.255
Contralat position	0.946	12	0.585
Cleft volume	0.933	12	0.408
Contralat volume	0.863	12	0.053
	**Non-Cleft Patients**		
	**W**	**df**	**Sig.**
Right height	0.910	14	0.157
Left height	0.934	14	0.342
Right width	0.961	14	0.733
Left width	0.896	14	0.100
Right depth	0.893	14	0.090
Left depth	0.958	14	0.687
Right position	0.923	14	0.246
Left position	0.945	14	0.487
Right volume	0.973	14	0.919
Left volume	0.959	14	0.710

**Table 2:** Patient characteristics of unilateral cleft lip and palate patients.

Age	Sex	Type	Cleft Side	Ocular	Osteotomy	Previous Operations
				pathology		
20 y 7 mo	M	UCLP	Left	No	Le Fort 1	Primary lip repair and soft palate closure 4 mo.
						Hard palate closure 11 mo.
						Alveolar bone graft 9 y 6 mo.
18 y 3 mo	M	UCLP	Right	Myopia	Bimaxillary	Primary lip repair 3 mo and soft palate closure 3 mo.
				astigmatism		Hard palate closure 9 mo.
						Alveolar bone graft 11 y 2 mo.
18 y 10 mo	M	UCLP	Left	No	Le Fort 1	Primary lip repair 4 mo. One-stage palatal closure 11 mo.
						VPI surgery (Furlow Z-plasty) 6 y 7 mo.
						Alveolar bone graft 10 y 10 mo. Early Le Fort 1 osteotomy 14 y 4 mo.
20 y 7 mo	M	UCLP	Left	No	Bimaxillary	Primary lip repair and soft palate closure 4 mo.
						Hard palate closure 11 mo. Secondary lip repair 6 y 9 mo.
						Secondary lip repair and rhinoplasty 11 y 9 mo. Alveolar bone graft 11 y 7 mo.
13 y 4 mo	F	UCLP	Left	No	Le Fort 1	Primary lip repair 3 mo. One-stage palatal closure 1 y 2 mo.
						Alveolar bone graft and fistula closure 9 y 2 mo.
12 y 5 mo	F	UCLP	Left	No	Le Fort 1	Primary lip repair 4 mo. One-stage palatal closure 8 mo.
						Fistula closure 5 y 4 mo. Alveolar bone graft 9 y 3 mo.
16 y 2 mo	F	UCLP	Left	No	Le Fort 1	Primary lip repair 3 mo. One-stage palatal closure 1 y.
						Previous operations abroad, no specific data.
						Alveolar bone graft 10 y 8 mo. Early Le Fort 1 osteotomy 13 y 3 mo.
16 y 2 mo	F	UCLP	Left	No	Le Fort 1	Primary lip repair and palatal closure around 1 y.
						Previous operations abroad, no specific data.
						Alveolar bone grafts 9 y 2 mo and 10 y 2 mo. Fistula closure 11 y 3 mo.
27 y 8 mo	F	UCLP	Left	No	Bimaxillary	Primary lip repair 3 mo. One-stage palatal closure 9 mo.
						Secondary lip repair 8 y 9 mo.
						Alveolar bone graft 9 y 1 mo. Rhinoplasty 17 y 7 mo.
18 y 2 mo	F	UCLP	Right	No	Le Fort 1	Primary lip repair and soft palate closure 3 mo. Hard palate closure 8 mo.
						VPI surgery (muscular repair of soft palate) 7 y 2 mo.
						Alveolar bone graft 16 y 7 mo.
18 y 10 mo	M	UCLP	Left	No	Le Fort 1	Primary lip repair and soft palate closure 3 mo. Hard palate closure 1 y.
						Fistula closure 5 y 6 mo.
						Alveolar bone graft 9 y 4 mo.
21 y 6 mo	M	UCLP	Left	No	Bimaxillary	Primary lip repair 3 mo. One-stage palatal closure 9 mo.
						VPI surgery (pharyngeal flap, Hogan) 5 y 6 mo.
						Alveolar bone graft 9 y 5 mo.
24 y 3 mo	M	UCLP	Right	No	Le Fort 1	Primary lip repair 3 mo. One-stage palatal closure 10 mo.
						Alveolar bone graft 10 y 3 mo.
16 y 8 mo	F	UCLP	Left	No	Bimaxillary	Primary lip repair 7 mo. One-stage palatal closure 1 y 1 mo.
						Alveolar bone graft 11 y 7 mo.
18 y 10 mo	M	UCLP	Right	No	Le Fort 1	Primary lip repair 3 mo. One-stage palatal closure 1 y.
						Alveolar bone graft 10 y 1 mo.
Mean 18, 82 y	8 M, 7 F		11 left, 4 right		10 Le Fort 1, 5 Bimax	

VPI surgery, surgery for velopharyngeal insufficiency; UCLP, unilateral cleft lip and palate; F, female; M, male; mo, months; y, years.

**Table 3:** Radiological measurements and comparisons of the cleft side and contralateral side.

	Cleft Side	Contralat. Side	Mean	95% CI	95% CI	DF	*p*-Value	
			Difference	Lower	Upper			
Height, cm								
*n* = 14	3.793	3.793	0	−0.0751	0.0751	13	1.00	NS
Width, cm								
*n* = 14	3.300	3.343	−0.0429	−0.0970	0.0113	13	0.111	NS
Depth, cm								
*n* = 15	4.520	4.567	−0.0467	−0.1326	0.0393	14	0.264	NS
Position, cm								
*n* = 15	6.660	6.888	−0.1733	−0.3129	−0.0338	14	0.019	*
Volume, mm^3^								
*n* = 12	25,596.825	26,433.167	−836.3417	−1464.8051	−207.8782	11	0.014	*

NS, non-significant. * *p* < 0.05, significant.

**Table 4:** Comparison of the bimaxillary and maxillary Le Fort 1 surgery subgroups of UCLP patients.

	Bimax, Cleft vs. Contralat. %	Le Fort 1, Cleft vs. Contralat. %	*p*-Value	
Height	101.56	99.04	0.518	NS
Width	98.82	98.72	0.898	NS
Depth	97.05	100.07	0.099	NS
Position	94.53	99.00	0.040	*
Volume	95.71	97.97	0.343	NS

NS, non-significant. * *p* < 0.05, significant.

**Table 5:** Radiological measurements of the non-cleft group (class III non-cleft patients).

	Right	Left	Mean	95% CI		DF	*p*-Value	
			Difference	Lower	Upper			
Height, cm								
*n* = 14	3.836	3.867	−0.0214	0.09	0.0472	13	0.512	NS
Width, cm								
*n* = 15	3.49333	3.507	−0.0113	−0.0545	−0.0545	14	0.499	NS
Depth, cm								
*n* = 16	4.919	4.875	0.0438	−0.0177	0.1052	15	0.15	NS
Position, cm								
*n* = 16	7.19375	7.169	0.025	−0.0465	0.0965	15	0.468	NS
Volume, mm^3^								
*n* = 14	24,591.429	24,448	143.571	−78.928	366.071	13	0.187	NS

NS, non-significant.

## Data Availability

Restrictions apply to the availability of these data. Data was obtained from Helsinki University Hospital Medical Archives and are available from the corresponding author with the permission of Helsinki University Hospital.
